# Progression of myocardial fibrosis by magnetic resonance imaging in patients with Duchenne and Becker muscular dystrophy and preserved left ventricular ejection fraction - a randomized clinical trial for treatment with ACE inhibitors

**DOI:** 10.1186/1532-429X-16-S1-P311

**Published:** 2014-01-16

**Authors:** Marly C Silva, Carlos H Rassi, Zilda M Meira, Juliana G Giannetti, Mariz Vainzof, Mayana Zatz, Roberto Kalil, Carlos E Rochitte

**Affiliations:** 1 Cardiology, Heart Institute, InCor, University of São Paulo Medical School, São Paulo, São Paulo, Brazil; 2 Cardiology and Pediatry, Federal University of Minas Gerais, Belo Horizonte, Minas Gerais, Brazil; 3 Human Genome Research Center, Bioscience Institute, University of São Paulo, São Paulo, São Paulo, Brazil; 4 Radiology, Axial Centro de Imagem, Belo Horizonte, Minas Gerais, Brazil

## Background

Duchenne (DMD) and Becker (BMD) muscular dystrophies( MD) are inherited X-linked diseases characterized by absence or decrease of dystrophin, a sarcolemal protein that is essential for maintenance of the muscular membrane integrity during muscular contraction. Cardiac involvement is as high, it can be clinically silent, but is often complicated by severe heart failure and high mortality. Angiotensin-converting enzyme inhibitors (ACEI) is recommended for patients with left ventricular dysfunction. We previously described that CMR can identify myocardial fibrosis (MF) even in the early stages of cardiomyopathy in MD before overt LV dysfunction (J Am Coll Cardiol 2007;49:1874-9). The impact of treatment with ACE inhibitors in the progression of fibrosis in patients with MD and preserved LV function is still unknown.

## Methods

We enrolled 76 pts, 70 pts with DMD and 6 BMD. Mean age was 13.1 ± 4.4 years. All patients underwent baseline and 2.3 years follow-up CMRs, using cine resonance for function evaluation and myocardial delayed enhancement (MDE) technique for MF detection. Forty-two pts with MF and normal LVEF were randomized into 2 groups, for receiving or not ACEI. CMR were performed on a 1.5-T Siemens Avanto (Erlangen, Germany). Two experienced observers measured LV volumes, ejection fraction and MF mass (5 standard deviation thresholding, on CMR42, v.3.4.2 Circle CVI, Calgary, Alberta, Canada). Wilcoxon test was used for comparison of MF between baseline and follow-up.

## Results

Two patients died before follow-up CMR. For all 74 patients, MF increased significantly from 20.8 ± 17.3 % to 26.6 ± 18.7 % on the follow-up, p < 0,001. In a sub-group with LV dysfunction at baseline (n = 11) MF increased from 31.6 ± 9.6 % to 40.6 ± 9.4 %, p = 0.013. Patients with MF and preserved LVEF that were randomized for treating with ACEI had lower evolution of MF than those who were randomized to untreated group (3.1 ± 7.4% vs. 10.0 ± 6.2%, respectively, p = 0.001). Lower progression of MF was also noted when comparing the two treated groups (LV dysfunction and normal LV function randomized to treated), 9.0 ± 9.9 % vs. 3.1 ± 7.4 %, p = 0,047, respectively. Using multivariate regression analysis, we found that belonging to the ACEI treated group decreases the progression of MF even after adjusting for age, creatine kinase level and baseline MF, p = 0.039) and indicated a trend for lower probability of presenting LVEF < 50% at follow-up CMR (OR = 3.18, p = 0.102, by logistic regression).

## Conclusions

Myocardial fibrosis, detected by CMR, progressively increases in all patients with Duchenne and Becker muscular dystrophy over a period of 2.3 years. In patients with MF and preserved LVEF, the treatment with ACEI decreases the progression of MF. Patients with LV dysfunction at baseline show progression of MF despite of ACEI therapy. Our data suggest that early initiation of ACEI therapy, before LV dysfunction can be detected, can decrease the progression of MF in DMD and BMD.

## Funding

No funding.

**Figure 1:**
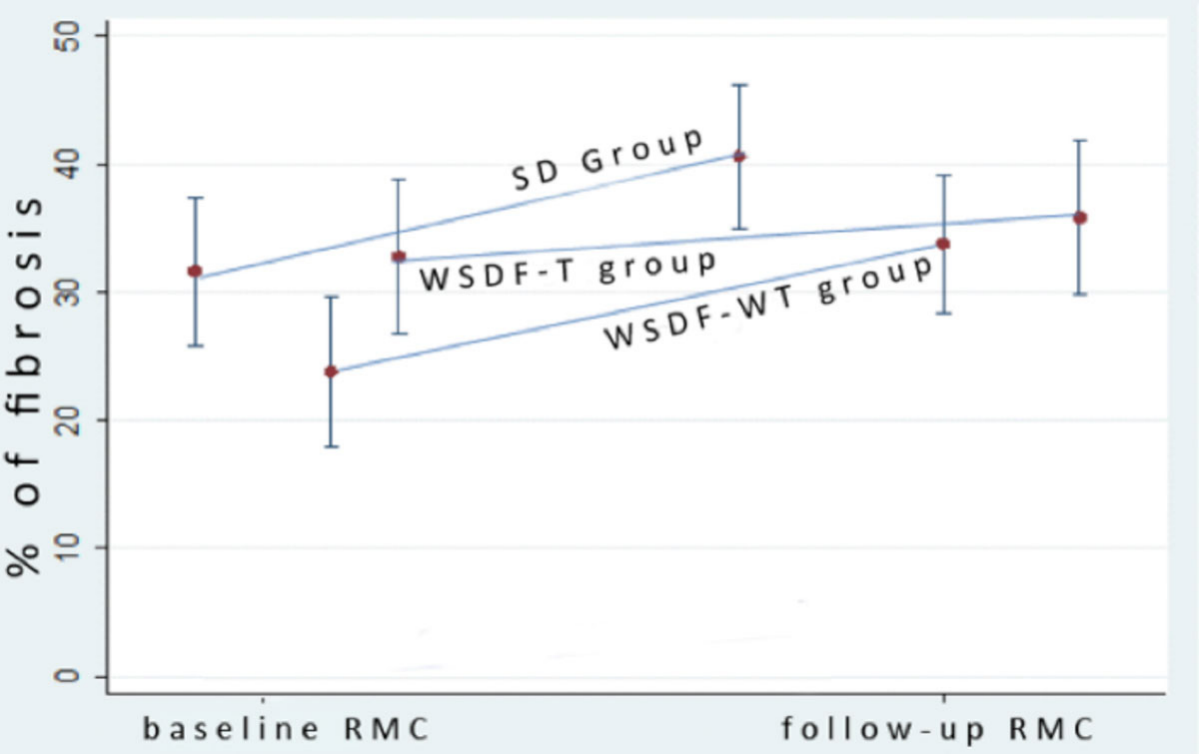
**Myocardial Fibrosis Progression in DMD and Becker pts over a period of 2.3 years**. SD Group - group with systolic dysfunction WSDF-T group - group without systolic dysfunction and with fibrosis, treated with ACEI WSDF-WT group - group without systolic dysfunction and with fibrosis, without treatment.

